# Acute Oral Toxicity and Anti-inflammatory and Analgesic Effects of Aqueous and Methanolic Stem Bark Extracts of *Piliostigma thonningii* (Schumach.)

**DOI:** 10.1155/2020/5651390

**Published:** 2020-08-06

**Authors:** Ben Olela, James Mbaria, Timothy Wachira, Gervason Moriasi

**Affiliations:** ^1^Department of Public Health, Pharmacology and Toxicology, College of Veterinary and Agricultural Sciences, University of Nairobi, P.O. Box 29053-00625, Nairobi, Kenya; ^2^Department of Biochemistry, Microbiology and Biotechnology, School of Pure and Applied Sciences, Kenyatta University, P.O. Box 43844-00100-G.P.O, Nairobi, Kenya; ^3^Department of Medical Biochemistry, College of Health Sciences, School of Medicine, Mount Kenya University, P.O. 342-01000, Thika, Kenya

## Abstract

Inflammation and pain are devastating conditions characterizing many diseases. Their manifestation ranges from mild body discomfort, to a debilitating experience, which may culminate in organ failure or death. In conventional medicine, corticosteroids, nonsteroidal anti-inflammatory drugs, opioids, and adjuvants are utilized to manage symptoms related to pain and inflammation. Despite their reported successes, these agents are only palliative, debatably inaccessible, unaffordable, and cause many undesirable side effects. As a result, the search for alternative and complementary therapies is warranted. Medicinal plants have been intensively utilized by humans for a long time to treat various ailments. In spite of their reported efficacies, empirical scientific data supporting their healing claims is scanty. *P. thonningii* (Schumach.) has been used in African traditional medicine, especially by traditional herbalists in Nigeria and Kenya, to treat conditions associated with inflammation. Even though analgesic, anti-inflammatory, and toxicity studies have been performed on leaf extracts, and some of their isolated compounds in Nigeria, there is scanty data supporting the use of stem bark extracts, which are commonly utilized in Kenya for pain, and inflammation management. Moreover, scientific data regarding safety and toxicity of the stem bark extracts of *P. thonningii* utilized in Kenya by traditional herbalists are inadequate. Based on this background, acute oral toxicity evaluation of the aqueous and methanolic stem bark extracts of *P. thonningii*, in Swiss albino mice, was performed according to the OECD/OCDE (2008) guidelines. Anti-inflammatory activities were investigated using the xylene-induced ear oedema in mice, whereas analgesic activities were examined following the acetic acid-induced writhing technique. The acute oral toxicity data was analyzed, and interpreted according to the OECDE (2008) guidelines. Anti-inflammatory and analgesic activities data were tabulated on MS Excel, and exported to GraphPad Prism (v8.3). Descriptive statistics were computed, and expressed as mean ± SEM. Thereafter, One-Way ANOVA followed by Tukey's test was performed. *p* < 0.05 was considered statistically significant. All the studied plant extracts had LD_50_ values > 2000 mg/kg bw, and were hence deemed to be nontoxic according to OECD/OCDE document no. 425. The results showed that the acetic acid-induced writhing frequency in mice administered the aqueous stem bark extract of *P. thonningii*, at a dose of 500 mg/kg bw, was not significantly different from that recorded for mice which received the reference drug (acetylsalicylic acid 75 mg) (*p* > 0.05). Additionally, at all the studied extract doses, significantly lower acetic acid-induced writhing frequencies were recorded in mice that received the aqueous stem bark extract of *P. thonningii*, compared with the writhing frequencies in mice that received the methanolic extract of the same plant (*p* < 0.05). On the other hand, the aqueous stem bark extract of *P. thonningii,* at doses of 100 mg/kg bw and 500 mg/kg bw, and the methanolic stem bark extract of the same plant, at a dose level of 500 mg/kg bw, exhibited significantly higher percentage inhibitions of xylene-induced oedema than the percentage inhibitions shown by the reference drug (dexamethasone 1 mg/kg bw) (*p* < 0.05). Generally, the aqueous stem bark extract of *P. thonningii*, at all the studied dose levels, caused significantly higher inhibitions of xylene-induced ear oedema in mice, compared with the percentage inhibitions shown by methanolic stem bark (*p* < 0.05). Therefore, the aqueous, and methanolic stem bark extracts of *P. thonningii*, grown in Kenya, possess peripheral analgesic and anti-inflammatory activities in Swiss albino mice. Hence, they have a potential of offering safe analgesic, and anti-inflammatory compounds. Further studies aimed at isolating, elucidating, and characterizing bioactive components from the studied extracts are recommended. Moreover, specific mode(s) through which these extracts exert the reported bioactivities should be established. Further toxicological investigations involving the studied plant extracts are encouraged to fully establish their safety.

## 1. Introduction

Inflammation is defined as a reaction to infection, irritation, or injury to tissues. It is characterized by five cardinal features, namely, tumor (swelling/oedema), color (redness), dolor (pain), fever (warmth), and *functio laesa* (organ/tissue dysfunction) [1]. These responses are indispensable in successful maintenance of body's homeostasis and pathogen eradication. The underlying goal of inflammatory events is localization and elimination of harmful assaults, and removal of damaged tissues, with their components, culminating in healing of injured tissues [2].

Chronic inflammation is among the major contributors of pathologic conditions burdening both the developed and the developing nations, especially in the African continent [3]. For instance, chronic inflammation is associated with the emergency and persistence of obesity-associated diabetes mellitus after insulin resistance, among a continuum of other complex human diseases [3–5].

Many pro- and anti-inflammatory mediators are secreted during inflammatory events and during assault [2, 3, 6]. However, some of these mediators, including interleukin-12 (IL-12), exhibit both proinflammatory and anti-inflammatory features [7]. Many of these mediators have been extensively demonstrated to play integral functions in human pathologic conditions. They include eicosanoids (prostaglandins and leukotrienes), cytokines (interferons, tumor necrosis-*α*, and, interleukins), chemokines (chemoattractant protein-1 and monocytes), and the nuclear factor kB transcription factor, which is a potent modulator of inflammation [3, 7].

Pain is an unpleasant sensory and emotional experience associated with potential/actual tissue damage [8]. It is a localized sensation ranging from mild discomfort to agonizing experience [9, 10]. Stimulation of nociceptors in the skin transmits pain messages to the brain for interpretation and response. Some nociceptors only respond to severe stimulations, while others respond to innocuous and warning stimuli [9, 10].

Ordinarily, treatment of algesia and inflammation depends on the use of nonsteroidal anti-inflammatory drugs (NSAIDs), adjuvants, and opioids. As noted by [11], most of the drugs are either expensive or inaccessible, and they often lead to adverse effects. Conventional drugs only provide a symptomatic relief, and they are often toxic to body tissues and organs, including the liver and kidney. In this regard, plant-based medicines, such as extracts from Piliostima thonningii (Schumach), can be used for treatment [3, 12]. This has necessitated the search for safer, affordable, and more effective alternatives to avert pain, inflammation and associated maladies. Natural products, especially those of plant origin, are more potent, easily accessible, affordable and are relatively less toxic compared to sythentic counterparts.

*Piliostigma thonningii* (synonym: *Bauhinia thonningii*) is legume of Caesalpinioideae family, growing up to 40 metres above the ground in tropical Africa [13]. Various parts of this plant are commonly used in many countries for managing various conditions including pain and inflammation [13–16].

Previous studies have demonstrated the anti-inflammatory, analgesic, and toxic effects of leaf extracts, and some compounds from leaves of *P. thonningii* in Nigeria [17–21]. However, there is no adequate scientific evidence to back the use of the stem bark extracts of *P. thonningii* in the management of pain and inflammation. To the best of our knowledge, there is no available scientific data supporting the analgesic and anti-inflammatory efficacy of the stem bark extracts of *P. thonningii* in Kenya, despite the continued ethnomedicinal applications for inflammatory disorders [16]. Additionally, the safety of the stem bark extracts of this plant, which are prominently utilized among traditional medical practitioners in Kenya, has not been determined.

Therefore, the present study aimed at investigating acute oral toxicity, anti-inflammatory, and analgesic effects of aqueous and methanolic stem bark extracts of *P. thonningii*, to provide empirical data for the discovery of well tolerable, affordable, safer, and accessible anti-inflammatory and analgesic drugs.

## 2. Materials and Methods

### 2.1. Plant Materials

Fresh barks of *P. thonningii* were obtained from Embu County, Kiang'ombe forest, in the natural habitat where the plant grew, with the help of a reputable local herbalist. Identification and authentication were done by a plant taxonomist at the East Africa Herbaria (National Museums of Kenya), and the plant was assigned the voucher reference number NMK/BOT/CTX/1/2. Duplicate voucher specimen was prepared and deposited at the Department of Biological Sciences, Chiromo Campus, University of Nairobi. The collected plant samples were cut into small fragments, air-dried in a well aerated room, with regular grabbling for 14 days, and ground into a coarsely powdered material using an electric mill. The powder was kept in well labelled manila bag and kept in a dry place awaiting extraction.

### 2.2. Extraction Methods

#### 2.2.1. Methanolic Extract

Extraction was performed according to the method described by Harborne [20] and modified by [21]. Briefly, 500 g of the powered material was macerated in 1 liter of analytical grade methanol in a 2-liter conical flask, and then covered with a foil paper with constant shaking for 48 h. The menstruum was decanted and filtered using a filter paper (Whatman No. 1). This procedure was repeated three times to exhaust extraction. The resultant filtrates were combined and reduced *in vacuo* at 55°C using a rotary evaporator. Thereafter, the extract was transferred into a clean, dry universal glass bottle, and placed in a hot-air oven set at 35°C, for complete drying. The extract was stored at 4°C in a refrigerator awaiting bioassay.

#### 2.2.2. Aqueous Extract

Approximately 100 g of the powdered plant materials was boiled in 750 ml of distilled water for a period of five minutes. The mixture was filtered through a filter paper (Whatman No. 1), cooled, and then lyophilized using a freeze-dryer *in vacuo*. The actual weight of the dried extract was measured using an analytical balance and recorded before it was stored in a refrigerator at 4°C awaiting biological assay [21].

### 2.3. Experimental Animals

In this study, Swiss albino mice (4-5 weeks old, weighing 24 ± 2 g) were obtained from the animal breeding facility of Public Health, Pharmacology and Toxicology Department, College of Veterinary and Agricultural Science, Kabete Campus of the University of Nairobi. The experimental animals were housed in polypropylene cages measuring 30 cm × 20 cm × 13 cm in standard laboratory conditions.

The bedding comprised soft wood shavings that were evenly spread in the holding cages to provide warmth to the housed animals and to deter dumping. Standard laboratory animal pellets and tap water were provided *ad libitum.* Animal use and care guidelines set out by the University of Nairobi Ethical Review Committee and the National Council for Science, Technology and Innovation (NACOSTI), were followed in this study.

### 2.4. Preparation of Administration Doses

Following a pilot study, doses of 4 mg/kg bw, 20 mg/kg bw, 100 mg/kg bw, and 500 mg/kg bw of the aqueous and methanolic stem bark extracts of *P. thonningii* were selected. To prepare appropriate dosages for administration to experimental mice, the OECD (2008, Document No. 425) guidelines illustrated by [22] were adopted. Briefly, to prepare a stock solution, of dose level 500 mg/kg bw, to be administered to a mouse weighing 20 g, the formula posited by [22] was followed as demonstrated:

(1) $$\begin{array}{c} \text{animal dose}\left(\text{mg}/\text{kg}\text{ bw}\right)=\frac{\text{body weight of the animal}\left(\text{g}\right)}{1000\text{ g}}\times \text{selected dose }\left[\text{15}\right]\text{,} \end{array}$$

so animal dose (mg/kg bw)  = 20(g)/1000 g × 500 mg = 10 mg.

According to the OECD [23] guidelines, 10 mg should be reconstituted in 0.2 ml of the vehicle (normal saline). In this study, a 10 ml stock solution containing 500 mg/kg bw of either the aqueous or the methanolic stem bark extracts of *P. thonningii* was prepared and serially diluted with normal saline to obtain 100 mg/kg bw, 20 mg/kg bw, and 4 mg/kg bw doses. The same procedure was followed for the standard drug (dexamethasone, 10 mg).

### 2.5. Acute Oral Toxicity Effects of the Aqueous and Methanolic Stem Bark Extracts of *P. thonningii*

To evaluate and appraise safety of the studied plant extracts, the Up-and-Down Procedure (UDP) for acute oral toxicity described by OECD [23] was adopted. The experimental mice were randomly selected and labelled with a permanent marker pen on their tails.

The mice were housed individually in polypropylene cages for 48 hours to help them acclimatize before being subjected to the study. Afterwards, an initial dose of 175 mg/kg bw was orally administered to the experimental group consisting of three (3) mice and 10 ml/kg bw of normal saline to the control group (3 mice).

Afterwards, wellness parameters including appearance of mucous membrane, eyes, skin fur, salivation, convulsions, lethargy, coma, sleep, diarrhea, tremors, body weight deviation, and mortality were monitored and recorded after 30 minutes, 1 hour, 4 hours, 24 hours, 48 hours, 7 days, and 14 days respectively [23]. The same procedure was adopted for a 550 mg/kg bw dose and for the cut-off dose of 2000 mg/kg bw [23].

### 2.6. *In Vivo* Anti-Inflammatory Effects of the Aqueous and Methanolic Stem Bark Extracts of *P. thonningii*

In this study, the xylene-induced ear oedema technique described by Igbe et al. [21] was adopted. Experimental mice were randomly divided into six groups (A, B, C, D, E, and F), with each group having five (5) mice. Mice in groups A, B, C, and D, respectively, were orally administered with 4 mg/kg bw, 20 mg/kg bw, 100 mg/kg bw, and 500 mg/kg bw of the studied plant extracts *p.o*. and 1 drop of xylene topically. The control groups (E and F, respectively) received 1 mg/kg bw of dexamethasone as positive control and 10 ml/kg bw of distilled water as negative control respectively, *p.o.* and 1 drop of xylene topically. The volume of administration was 200 *μ*l for all the agents except for xylene which was administered by smearing 1 drop of xylene on the inner pinna of the right ear.

After 60 minutes, oedema in each mouse was induced by smearing 1 drop of xylene on the inner pinna of the right and left ears for 15 minutes. Afterwards, the experimental mice were anesthetized using diethyl ether and both the right (oedematous) and left ears were dissected and accurately weighed using an analytical balance. The respective weights were recorded and used to calculate the anti-inflammatory effects of the extracts and expressed as the percentage inhibition of oedema according to the formula described by Igbe et al. [21]:

(2) $$\begin{array}{c} \%\text{ inhibition of edema}=\frac{A-B}{A}\times 100, \end{array}$$

where *A* is the difference in ear weight in the negative control and *B* is the difference in ear weight in the experimental/positive control mice.

### 2.7. Determination of the Analgesic (Antinociceptive) Activity of the Aqueous and Methanolic Stem Bark Extracts of *P. thonningii*

Peripheral analgesic effects of the aqueous and methanolic stem bark extracts of *P. thonningii* were evaluated using the acetic acid-induced writhing method of [24] in Swiss albino mice. A completely randomized study design was adopted, from which the experimental design was drawn. In this design, experimental mice were randomly assigned to six groups (I, II, III, IV, V, and VI), with each consisting of 5 animals. Groups I, II, III, and IV received an oral treatment of 4 mg/kg bw, 20 mg/kg bw, 100 mg/kg bw, and 500 mg/kg bw, respectively, of the studied plant extracts *p.o*. On the other hand, groups V and VI received 75 mg/kg bw of acetylsalicylic acid (Aspirin) and 10 ml/kg bw of distilled water orally as positive and negative controls, respectively. After 30 minutes, writhing was induced in each experimental mouse with an intraperitoneal injection of 0.6% v/v acetic acid. All the drugs were administered at a volume of 200 *μ*l.

Thereafter, experimental mice were monitored individually, and the number of writhes was counted after 5 minutes of writhing induction, for 30 minutes, and recorded. The average number of writhes and the percentage inhibition of writhing were calculated as an indicator of analgesic activity following the equation described by Rashid et al. [24]:

(3) $$\begin{array}{c} \%\text{ writhing inhibition}=\frac{\text{Wc}-W}{\text{Wc}}\times 100, \end{array}$$

where Wc is the mean number of writhes in the control and *W* is the mean number of writhes in the experimental group (extracts/standard).

### 2.8. Statistical Data Management and Analysis

The obtained data from anti-inflammatory and analgesic activities were tabulated on MS Excel spreadsheet (2016) and exported to GraphPad Prism statistical software version 8.3.0.538. The data were subjected to descriptive statistics and expressed as mean ± standard error of the mean (SEM) of replicate experiments. One-Way ANOVA was done to compare differences among means, followed by Tukey's *post hoc* test for pairwise comparison and separation of means at *α* = 0.05. Values with *p* ≤ 0.05 were considered statistically significant. Acute oral toxicity data were qualitatively and quantitatively analyzed according to OECD guideline document No. 425 [23], and LD_50_ values were recorded.

### 2.9. Ethical Considerations

Permission to conduct this study was obtained from University of Nairobi Ethical Committee and the National Council of Science Technology and Innovation (NACOSTI) under licence number NACOSTI/P/19/2442.

## 3. Results

### 3.1. Acute Oral Toxicity Effects of the Studied Plant Extracts

Acute oral toxicity effects of the aqueous and methanolic stem bark extracts of *P. thonningii* were also investigated in this study. The results demonstrated no observable signs of toxicity and lethal effects in experimental groups of mice even at the limit/cut-off dose of 2000 mg/kg bw. The LD_50_ values for each of the studied plant extracts were thus envisaged to be above 2000 mg/kg bw.

### 3.2. *In Vivo* Anti-Inflammatory Effects of the Aqueous and Methanolic Stem Bark Extracts of *P. thonningii*

**Figure 1:**
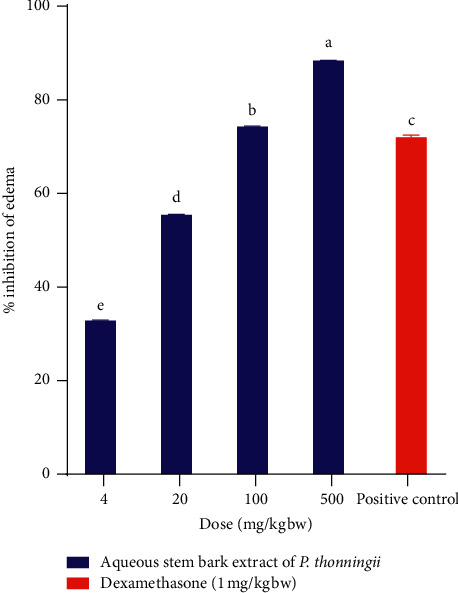
Effect of the aqueous stem bark extract of *P. thonningii* on xylene-induced ear oedema in mice. Values are plotted as mean ± SEM. Bars with different superscript letters are significantly different (One-Way ANOVA followed by Tukey's test; *p* < 0.05).

The obtained results showed significant reductions in xylene-induced ear oedema in mice. The experimental mice that received 4 mg/kg bw of the aqueous stem bark extract of *P. thonningii* showed a significantly lower percentage inhibition of xylene-induced ear oedema in mice, compared with the percentage inhibition produced by the standard drug (dexamethasone) (*p* < 0.05; Figure 1).

Generally, a dose-dependent increase in percentage inhibition of xylene-induced ear oedema, caused by this extract was noted among the studied doses. Remarkably, the aqueous stem bark extract of *P. thonningii* at a dose of 500 mg/kg bw revealed the highest percentage inhibition, compared with the other dose levels of the same extract and the standard drug (*p* < 0.05; Figure 1).

**Figure 2:**
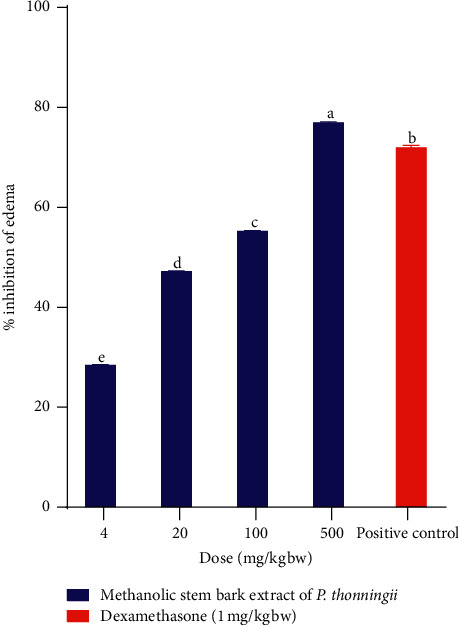
Effect of the methanolic stem bark extract of *P. thonningii* on xylene-induced ear oedema in mice. Values are plotted as mean ± SEM. Bars with different superscript letters are significantly different (One-Way ANOVA followed by Tukey's test; *p* < 0.05).

Similarly, the results revealed that the methanolic stem bark extract of *P. thonningii* at dose levels of 4 mg/kg bw, 20 mg/kg bw, and 100 mg/kg bw exhibited significantly lower percentage inhibition of xylene-induced ear oedema in experimental mice, compared with the percentage inhibition in mice treated with dexamethasone at a dose of 1 mg/kg bw (*p* < 0.05; Figure 2).

However, the results showed that the methanolic stem bark extract of the studied plant at a dose of 500 mg/kg bw significantly inhibited xylene-induced ear oedema in mice, more than the inhibitions caused by all the other dose levels of the same extract and dexamethasone (*p* < 0.05; Figure 2). Overall, a positive dose-dependent increase in percentage inhibition of oedema was observed in the experimental mice that received the studied plant extract (Figure 2).

**Figure 3:**
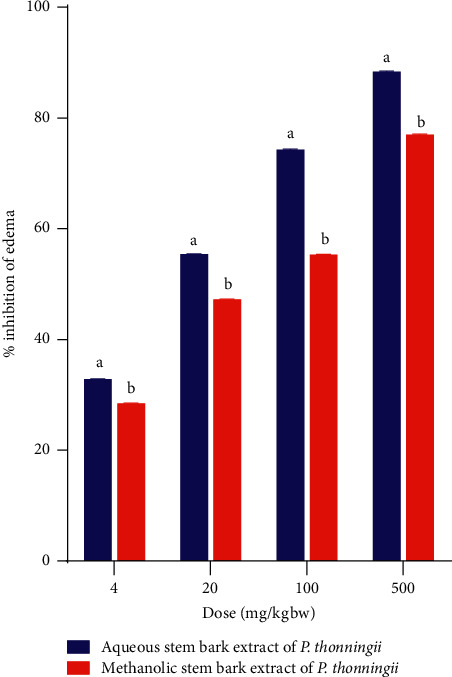
Comparison between the effects of the studied plant extracts on xylene-induced ear oedema. Values are plotted as mean ± SEM. Bars with different superscript letters within the same dose level are significantly different (Un-paired Student's *t*-test; *p* < 0.05).

Moreover, a comparison between the effects of the aqueous and methanolic stem bark extracts of *P. thonningii* in xylene-induced ear oedema was done. The results showed that the experimental mice administered the aqueous stem bark extract of this plant, at all the studied dose levels, showed significantly higher percentage inhibitions of oedema compared with the inhibitions in mice that received the methanolic extract at all the studied dose levels (*p* < 0.05; Figure 3).

### 3.3. *In Vivo* Analgesic Effects of the Aqueous and Methanolic Stem Bark Extracts of *P. thonningii*

In this study, the results revealed a dose-dependent reduction in writhing corresponding to an increase in parentage inhibition of acetic acid-induced writhing in mice (Table 1). Upon administration of the aqueous stem bark extract of *P. thonningii* to mice, the percentage inhibition of writhing significantly increased in a dose-dependent manner (*p* < 0.05; Table 1).

Notably, at a dose level of 500 mg/kg bw, the mice that received the aqueous stem bark extract of *P. thonningii* showed significantly reduced writhing, compared to that recorded for mice in all the other experimental groups including those in the positive control group (*p* < 0.05; Table 1).

On the other hand, the mice that received the methanolic stem bark extract demonstrated significantly reduced writhing in a dose-dependent fashion (*p* < 0.05; Table 2). The results indicated significantly higher writhing frequency in mice treated with 4 mg/kg bw compared with the writhes in mice that received the other studied extract doses and the standard drug (*p* < 0.05; Table 2).

Conversely, the experimental mice that were administered this extract at a dose level of 500 mg/kg bw demonstrated significant lower writhing frequency, compared with the writhing frequencies recorded for mice in all the other experimental groups (*p* < 0.05; Figure 2). Interestingly, at a dose level of 500 mg/kg bw of the methanolic stem bark extract of *P. thonningii*, the recorded number of abdominal writhes was significantly lower than the writhes in the positive control (*p* < 0.05; Table 2).

**Figure 4:**
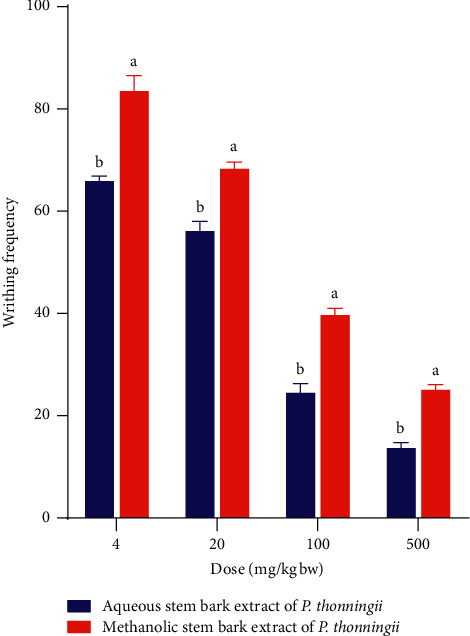
Comparison between the effects of the studied plant extracts on acetic acid-induced writhing in mice. Values are plotted as mean ± SEM. Bars with different superscript letters within the same dose level are significantly different (Un-paired Student's t-test; *p* > 0.05).

A comparison of the effects of the studied plant extracts on acetic acid-induced writhing in mice was also done in this study (Figure 4). The results revealed that, at all the studied dose levels, the aqueous stem bark extract of *P. thonningii* conferred significantly higher percentage inhibition (less writhing frequency) of acetic acid-induced writhing compared to its methanolic counterpart at the same dose levels (*p* < 0.05; Figure 4).

## 4. Discussion

Inflammation is the wellspring of symptoms elicited by maladies affecting the body. It is a multifaceted biological response by vascular tissues to injurious stimuli like pathogens, damaged cells, physical and chemical assaults, as well as immunological responses [25]. As far as humankind and health are concerned, understanding of inflammation and associated processes has been a major conundrum [26].

The cardinal signs which characterize inflammation include increased cellular metabolism, release of cellular soluble inflammatory mediators, increased blood flow, vasodilation, extravasation of fluids and cellular influx, formation of abnormal granulations, necrosis, and excessive tissue degeneration and exudation. All these lead to varying degrees of tissue injuries and can even lead to death [1].

In this study, acute inflammation was evaluated using the xylene-induced ear oedema technique. Xylene is a chemical inflammatory trigger which causes release of inflammatory mediators including histamine, bradykinin, and serotonin [27]. Consequently, there is greater permeability in the vasculature with elevated vasodilation. The result is the accumulation of fluid at the inflamed/induced site observed in the xylene-induced ear oedema in mice [27].

A drug agent that is capable of inhibiting fluid accumulation in this technique is considered as having anti-inflammatory activity [27]. The results reported herein suggest remarkable anti-inflammatory effects of the aqueous and methanolic stem bark extracts of *P. thonningii* as evidenced by their ability to inhibit/reduce xylene-induced ear oedema in experimental mice. Particularly, the aqueous stem bark extract of *P. thonningii* proved to be more potent than the methanolic extract of the same plant, at all the studied dose levels.

The current medications used to avert inflammation are topical steroids and nonsteroidal anti-inflammatory drugs which work by inhibiting the activity of phospholipase A_2_ [5, 28, 29]. It is suggestive that partly the studied plant extracts could be working through this mechanism. Moreover, research has established that peripheral action differs from central action, and some centrally acting drugs, such as opioids, inhibit both phases of nociception equally [30]. Even though the specific mechanism through which the studied plant extracts exert their bioactivity has not been debunked, it is suggestive that the compounds in these extracts could possess both peripheral and central analgesic/anti-inflammatory effects.

Generally, acetic acid-induced writhing test serves as a standard technique for evaluating antinociceptive/analgesic efficacy of natural products [31, 32]. In this method, acetic acid induces the release of various endogenous noxious mediators such as histamine, serotonin, and bradykinin which drive the cardinal signs associated with inflammation, especially pain [27]. The pain brought by the acetic acid is evidenced in the contraction of abdominal muscles and the expansion of forelimbs, as well as body elongation, that is generally regarded as writhing, whose frequency is quantified in 30 minutes [7, 27]. This elongation is thought to be caused by the activation of local peritoneal receptors and prostaglandin pathways in the experimental model animal, by appropriate chemicals like acetic acid [33].

Upon injection of acetic acid intraperitoneally into experimental mice, it causes the release of inflammatory mediators which excites pain receptors (nociceptors), which in turn send pain messages to the central nervous system and the brain through the prostaglandin system [34]. Furthermore, other pain mediators like bradykinins and histamine are released from cells lining the peritoneal cavity and further help stimulate nociceptors [9, 35]. Agents which reduce/inhibit the acetic acid-induced writhing frequency are considered as having analgesic effects.

In this study, the studied stem bark extracts of *P. thonningii* plant showed significant inhibition of the acetic acid-induced writhing in a manner that suggests blockade/inhibition of the prostaglandin pathway in the pain perception cascade. Previous studies have shown that drugs which inhibit the cyclooxygenase enzyme pathway inhibit writhing, which is an indicator of pain in experimental animal models [36].

The findings of this study agree with those reported in a previous study; that is, NSAIDs reduce the number of writhes by inhibiting cyclooxygenase in peripheral tissues [36]. The aqueous and methanolic stem bark extracts of the studied plant may be acting through a similar mechanism in averting pain in this study. Indeed, the findings of this study are consistent with previous studies which indicated that the aqueous leaf extract of *P. thonningii* possess demonstrable analgesic activity in the writhing test [21].

Further, anti-inflammatory compounds have been isolated from the leaf extracts of *P. thonningii* growing in Nigeria [18]. Among the isolated compounds, 6-C-methylquercetin 3,7,3′-trimethyl ether (3) proved to be a potent prostaglandin synthesis inhibitor, indicating a promising potential of this plant as a source of potent anti-inflammatory agents [18].

It has been demonstrated that oxidative stress plays a role in inflammatory processes, and that antioxidant compounds play major roles in amelioration of inflammation [37]. Preliminary phytochemical investigations of aqueous and methanolic stem bark extracts of *P. thonningii* have shown presence of flavonoids and phenols among other antioxidant phytocompounds [41]. Further, an earlier study by Kwaji et al. [42] on the aqueous leaf extract of *P. thonningii* showed presence of antioxidant phytochemicals which are associated with anti-inflammatory activity [41]. It is therefore anticipated that the analgesic and anti-inflammatory effects of the studied extracts could be due to these secondary metabolites.

Although most of drugs presently used to manage pain and inflammation are effective, they have been associated with toxic effects and adverse side effects as noted by Deghrigue et al. [40]. As a result, there is a continuous search for alternative treatments that can alleviate these conditions with minimum adverse events. Herbal medicines have been established to be a safe alternative due to their natural origin, cultural adaptability, availability, and safety [42].

Despite the utilization of herbal preparations in traditional medicine for a long time by humans to manage a variety of diseases, their safety and toxicity have raised concerns [43]. This has been exacerbated by the lack of clear guidelines and policies on traditional medicine practice, dosage regimes, formulations of herbal preparations, and little understanding of modes of action and drug interactions as well as toxicity profiles [44, 45]. As a result, it is imperative to investigate the safety of medicinal plants used in traditional medicine to provide essential scientific data, which can guide the validation of their claimed healing efficacies [46, 47].

In this study, acute oral toxicity effects of both the methanolic and aqueous stem bark extracts of *P. thonningii* were investigated according to the guidelines described by the OECD [23]. There were no observable signs of toxicity in the experimental group mice at the various dose levels, up to the cut-off dose of 2000 mg/kg bw. Thus, the study agrees with previous findings, such as those by [48, 49] which had found acute toxicity to be very low and that the extract is practically nontoxic at oral doses. Furthermore, [50] reported that the leaf fraction of *P. thonningii* was not toxic to rats at oral doses.

These results indicate that the aqueous and methanolic stem bark extracts of this plant are nontoxic to laboratory rodents. Studies have shown that the presence of toxic secondary metabolites is responsible for the adverse side effects reported when some plants or their products have been consumed [51]. Indeed, based on the results herein, the studied plant extracts, either lack the toxic amalgams, or are present in too low levels to cause any observable signs of toxicity. Therefore, these results partly support the traditional use of the studied plant extracts in the management of pain, and inflammation, among other claimed conditions [14, 16, 42, 52]. However, further studies are imperative to fully establish their safety and dosage formulations, as well as to understand drug interaction effects.

## 5. Conclusions and Recommendations

Based on the results obtained in this study, it was concluded that the aqueous and methanolic stem bark extracts of *P. thonningii* have acute anti-inflammatory and analgesic effects in Swiss albino mice. Furthermore, the aqueous and methanolic stem bark extracts of *P. thonningii* have LD_50_ values of >2000 mg/kg bw and are thus nontoxic as per the guidelines of OECD [23]. Therefore, the aqueous and methanolic stem bark extracts of *P. thonningii* can be utilized as alternatives in the management of inflammation and pain, as claimed in traditional medicine.

Nevertheless, there is a need to bio-screen the studied extracts of *P. thonningii* to identify and isolate the specific compounds with analgesic and anti-inflammatory activities. This way, new compounds might be discovered, which will be used for treatment of the two conditions: pain and inflammation. Additionally, future studies should focus on elucidation of the possible mechanism(s) for analgesic and anti-inflammatory actions of the aqueous and methanolic stem bark extracts studied herein. In addition to acute toxicity, there is a need to evaluate chronic toxicity to determine the safety of the bark extracts in animal models.

## Figures and Tables

**Table 1:** Effects of the aqueous stem bark extract of *P. thonningii* on acetic acid-induced writhing in mice.

Dose (mg/kg bw)	Aqueous stem bark extract of *P. thonningii*	
	Writhing frequency	% inhibition
4	66.20 ± 1.16^b^	29.72
20	56.40 ± 2.04^c^	40.13
100	24.80 ± 1.86^d^	73.67
500	14.00 ± 1.14^e^	85.14
Acetylsalicylic acid (75 mg/kg bw)	15.40 ± 1.21^e^	83.65
Negative control	94.20 ± 3.97^a^	0

Values are presented as mean ± SEM; means with different superscript letters along the column are significantly different (One-Way ANOVA followed by Tukey's test; *p* < 0.05).

**Table 2:** Effect of the methanolic stem bark extract of *P. thonningii* on acetic acid-induced writhing in mice.

Dose (mg/kg bw)	Methanolic stem bark extract of *P. thonningii*	
	Writhing frequency	% inhibition
4	83.80 ± 3.14^b^	11.04
20	68.60 ± 1.44^c^	27.18
100	40.00 ± 1.41^d^	57.54
500	25.40 ± 1.12^e^	73.04
Acetylsalicylic acid (75 mg/kg bw)	15.40 ± 1.21^f^	83.65
Negative control	94.20 ± 3.97^a^	0

Values are presented as mean ± SEM; means with different superscript letters along the column are significantly different (One-Way ANOVA followed by Tukey's test; *p* < 0.05).

## Data Availability

All data are available within the manuscript, and additional data are available from the corresponding authors on request.
